# Medicare Enrollment and Spending Among Patients Initiating Dialysis After the Affordable Care Act

**DOI:** 10.1001/jamahealthforum.2024.4304

**Published:** 2024-12-06

**Authors:** Virginia Wang, Lauren E. Wilson, Neil P. Rowen, Caroline E. Sloan, Matthew L. Maciejewski, Bradley G. Hammill

**Affiliations:** 1 Department of Population Health Sciences, Duke University School of Medicine, Durham, North Carolina; 2 Department of Medicine, Duke University School of Medicine, Durham, North Carolina; 3 Margolis Institute for Health Policy, Duke University, Durham, North Carolina; 4 Durham Center of Innovation to Accelerate Discovery and Practice Transformation, Durham Veterans Affairs Health Care System, Durham, North Carolina; 5 Kaiser Permanente Bernard J. Tyson School of Medicine, Pasadena, California

## Abstract

**Importance:**

Medicare finances health care for most US patients with end-stage kidney disease (ESKD), regardless of age. Medicare enrollment may have slowed for patients with incident ESKD who gained access to new private insurance options with the 2014 passage of the Affordable Care Act (ACA) and introduction of the ACA Marketplace.

**Objective:**

To describe trends in public and private insurance coverage and dialysis spending among patients with incident ESKD from 2012 to 2017.

**Design, Setting, and Participants:**

This serial cross-sectional study included patients 18 to 64 years old in Colorado who were not enrolled in Medicare at dialysis initiation. Data analysis was conducted from May to August 2023.

**Exposure:**

Introduction of the ACA Marketplace in 2014.

**Main Outcomes and Measures:**

Medicare, Medicaid, or private insurance enrollment in the first year after dialysis initiation, and dialysis spending by insurance type.

**Results:**

Of 2005 patients included in the sample, 1416 (70.6%) were 45 to 64 years old, and 1259 (62.8%) were male. A lower proportion of patients with incident ESKD starting dialysis were newly enrolled in Medicare in the years following the ACA (361 of 713 [50.6%]) compared to 2 years prior (420 of 595 [70.6%]). Unadjusted rates of switching from Medicaid to Medicare 1 year after dialysis initiation decreased 14.3 percentage points over time (68.9% in 2012-2013 vs 58.3% and 54.6% in 2014-2015 and 2016-2017, respectively). Unadjusted rates of switching from private insurance to Medicare 1 year after dialysis initiation decreased by 22.3 percentage points (68.1% in 2012-2013 vs 52.2% and 45.8% in 2014-2015 and 2016-2017, respectively). Over the entire 2012 to 2017 period, quarterly dialysis spending in the first year of dialysis among patients with private insurance was higher than among those with Medicare coverage ($26 351-$29 781 vs $10 039-$12 741).

**Conclusions and Relevance:**

This cross-sectional study demonstrates that lower Medicare enrollment rates over time among those initiating dialysis may be inducing higher social spending. This finding raises concerns about the effectiveness of Medicare policies and federal leverage to improve access, outcomes, and value of dialysis care.

## Key Points

**Question:**

Did new Medicare enrollment among patients undergoing dialysis in Colorado decline with the introduction of the Affordable Care Act Marketplace, and did dialysis spending differ across public and private payers?

**Findings:**

In this cross-sectional study of 2005 patients with end-stage kidney disease who were not actively enrolled in Medicare when initiating dialysis, Medicare enrollment declined among patients younger than 65 years initiating dialysis from 2012 to 2017. Annual dialysis spending was higher for privately vs publicly insured patients.

**Meaning:**

Fewer patients undergoing dialysis are enrolling in Medicare and instead choosing private insurance, which warrants more comprehensive data to investigate private insurance payment arrangements and the impacts of shifts in payer mix on patient care and national spending.

## Teaser

This cross-sectional study describes trends in public and private insurance coverage and dialysis spending among patients with incident end-stage kidney disease from 2012 to 2017.

## Introduction

Medicare has been the dominant payer for dialysis in the US since the 1972 passage of near-universal coverage for end-stage kidney disease (ESKD).^1^ Consequently, dialysis facilities have historically been sensitive to Medicare payment policies.^2^ Additionally, Medicare provides lower annual reimbursement than private payers ($80 500 vs $238 000), creating incentives for dialysis facilities to preferentially serve privately insured patients.^3^ Medicare enrollment declined among patients receiving dialysis after Medicare reduced reimbursement for hemodialysis in 2011^4^ and declined further after the 2014 Affordable Care Act (ACA) expanded access to private insurance coverage and, for some states, Medicaid.^5^ One reason for declining Medicare enrollment among patients receiving dialysis may be that dialysis facilities encourage patients to maintain private insurance. Historically, it has been difficult to examine whether declining Medicare enrollment is due to retention of private insurance, Medicaid, or other coverage because national disease registry data lack detail on non-Medicare insurance.

In this study, we address this measurement issue by conducting, to our knowledge, the first linkage of national health registry and claims data from the Colorado All Payer Claims Database (CO APCD) to examine trends in Medicare, Medicaid, and private insurance enrollment for patients initiating dialysis before and after passage of the ACA (2012-2017). We also leverage these linked data to describe trends in dialysis spending over time across payer types to provide a better understanding of the implications of public and private insurance enrollment trends on access and costs. Declining Medicare enrollment and differences in dialysis payments between Medicare and other payers raise important questions about how effective future Medicare dialysis policy changes can improve access to dialysis services, quality of dialysis care, and cost containment.

## Methods

### Study Design, Data Sources, and Sample

In a serial cross-sectional study, we linked the US Renal Data System (USRDS),^6^ the national registry of patients with ESKD and claims data from the CO APCD administered by the Center for Improving Value in Health Care and the Area Health Resource Files. The USRDS contains information on patients with ESKD, including Medicare insurance status and demographic and clinical characteristics. The CO APCD contains longitudinal enrollment and claims history for patients enrolled in Medicare fee-for-service, Medicare Advantage, Medicaid, and private insurance (eg, state exchange, individual, group coverage), and enables construction of the outcomes of insurance enrollment and dialysis spending (eg, dialysis facility charges, insurer reimbursement, and patient payments). The CO APCD contains records of patients’ insurer/payer in the APCD reporting period, so patients without documented insurance from some source were defined as uninsured. Area Health Resource Files county-level general population demographics were aggregated to generate market-level (hospital service areas [HSAs])^7^ statistics for each study year. The sample included all patients in the linked data residing in Colorado who were 18 to 64 years old (below threshold for age-eligible Medicare enrollment) with a new diagnosis of ESKD (“incident ESKD”) initiating dialysis treatment between 2012 and 2016, with 1-year follow-up through 2017. We focused on patients starting dialysis because temporal shifts in insurance coverage are most observable among those newly eligible for Medicare coverage due to ESKD onset. The pre-ACA policy period comprised years 2012 and 2013. The first postpolicy period reflected the initial years during which both ACA’s Medicaid expansion and Marketplace were implemented (2014-2015), and the second postpolicy period comprised the later ACA years (2016-2017). Patients were excluded if they had missing or delayed submission of demographic information (n = 683), were 65 years or older, or were actively enrolled in Medicare at the time of dialysis initiation (n = 3691). The final sample consisted of 2005 patients (eFigure 1 and eTable 1 in Supplement 1).

The study protocol was approved by the institutional review board at Duke University, and patient informed consent was waived owing to use of retrospective, deidentified information. This study followed the Strengthening the Reporting of Observational Studies in Epidemiology (STROBE) reporting guidelines.

### Measures

There were 2 outcomes of interest. The first outcome was Medicare enrollment 1 year after dialysis initiation, defined as being actively enrolled in Medicare Advantage or traditional Medicare as either the primary or secondary payer. Historically, most new patients with kidney failure make decisions about Medicare enrollment at either ESKD onset or dialysis initiation (ie, day 1 of dialysis).^5^ Patients have the option to apply later, and nearly all do so within the first year of initiating maintenance dialysis for various reasons, including emergency or urgent dialysis initiation and loss of other sources of coverage that were present at the time of diagnosis. Most individuals with ESKD applying for Medicare are required to undergo a 90-day waiting period before enrollment is activated (thus, actively enrolled in Medicare by the end of their first year receiving dialysis) (eFigure 2 in Supplement 1). This waiting period is waived for patients initiating home dialysis. The second outcome of interest was dialysis spending, operationalized as total dollars per day paid by third parties (ie, insurers) and patients (ie, cost share) for any services on days with an outpatient claim for dialysis, billed by organizations providing dialysis.

There were 2 explanatory variables of interest. The first was a 3-category variable of time of dialysis initiation: (1) before ACA Marketplace implementation (pre-ACA period, 2012-2013), (2) initial post-ACA period (2014-2015), and (3) later post-ACA period (2016-2017). The second explanatory variable of interest was an insurance coverage at dialysis initiation. Non-Medicare forms of insurance coverage included Medicaid, private, no documented medical insurance (eg, insurer not participating in CO APCD reporting, patient with only dental coverage), and other insurance (eg, patient had an enrollment record in the CO APCD but no claim for dialysis treatment). Due to small sample sizes, categories of “other insurance” and “no documented medical insurance” were combined.

Characteristics that may influence Medicare enrollment were constructed for descriptive and regression purposes to account for differences in the sample’s composition across the 3 time periods, including age, sex, race (Black, White, and other [American Indian or Alaska Native, Asian, Hawaiian or Pacific Islander, and other race; grouped together due to small sample sizes]), Hispanic ethnicity, employment status, cause of ESKD, receipt of pre-ESKD nephrological care, baseline clinical characteristics (kidney function,^8^ body mass index, and comorbid conditions [eTable 1 in Supplement1]). HSA region-level characteristics included general population per-capita income and dialysis facility competition,^9,10,11^ based on the number of patients undergoing dialysis unique to each facility.^12^

### Statistical Analysis

We describe unadjusted trends in insurance coverage in the first year following dialysis initiation for the entire sample of 2005 patients who were not enrolled in Medicare at dialysis initiation. Follow-up time started at dialysis initiation, and patients were censored at the earliest of (1) 1 year after dialysis initiation or (2) end of data availability (December 31, 2017). In adjusted analyses, a Poisson model with robust error estimates was used to estimate the relative risk of new Medicare enrollment at the end of the first year of dialysis, as a function of ACA policy periods, patient demographics, clinical characteristics, and HSA market characteristics.

In a descriptive analysis of dialysis spending in the first year following dialysis initiation, we examined the subset of 1095 patients who had an available claim for their first dialysis encounter in either the CO APCD or USRDS Medicare fee-for-service files. Means and IQRs of expenditures were aggregated per quarter and reported, with insurance status for each patient ascertained at the start of each quarter. If a patient died or had follow-up time end prior to the end of 1 year, they were not included in the cost calculations in the quarter following the censoring event. Next, annual dialysis spending was calculated for each patient by patterns of insurance coverage throughout the first year following dialysis initiation because many patients changed coverage sources in the first year. A 2-sided significance threshold was set at *P* < .05. Analysis was conducted from May to August 2023 using SAS, version 9.4 (SAS Institute).

## Results

### Patient Characteristics at Dialysis Initiation

Among the sample’s 2005 patients who were not actively enrolled in Medicare when initiating dialysis in 2012 to 2017, 1416 (70.6%) were 45 to 64 years old; 1259 (62.8%) were male; 242 (12.1%) were Black, 1619 (80.7%) were White, and 144 (7.2%) were of another race; 592 (29.5%) were of Hispanic ethnicity; and 1306 (65.1%) had received nephrological care before ESKD onset (Table 1). Among the patients, 639 (31.9%) were employed part or full time. Most patients new to dialysis lived in urban, regional markets with market competition and a high presence of organizations providing dialysis (median [IQR] Herfindahl-Hirschman Index value, 0.2 [0.0-0.4]). These characteristics were similar across the 3 policy periods (Table 1).

**Table 1.** Baseline Characteristics of Patients Not Enrolled in Medicare at Onset of Kidney Failure by Dialysis Initiation Year

Characteristic	No. (%)				*P* value
	Overall (N = 2005)	2012-2013 (n = 595)	2014-2015 (n = 697)	2016-2017 (n = 713)	
New Medicare enrollment at first dialysis	1043 (52.0)	392 (65.9)	339 (48.6)	312 (43.8)	<.001
Insurance at dialysis initiation					
Medicaid	883 (44.0)	209 (35.1)	319 (45.8)	355 (49.8)	<.001
Private	369 (18.4)	91 (15.3)	134 (19.2)	144 (20.2)	
Other/no documented insurance^a^	753 (37.6)	295 (49.6)	244 (35.0)	214 (30.0)	
Age, y					
18-44	589 (29.4)	169 (28.4)	195 (28.0)	225 (31.6)	.049
45-54	597 (29.8)	201 (33.8)	195 (28.0)	201 (28.2)	
55-64	819 (40.8)	225 (37.8)	307 (44.0)	287 (40.3)	
Sex					
Female	746 (37.2)	210 (35.3)	262 (37.6)	274 (38.4)	.49
Male	1259 (62.8)	385 (64.7)	435 (62.4)	439 (61.6)	
Race					
Black	242 (12.1)	74 (12.4)	85 (12.2)	83 (11.6)	.99
White	1619 (80.7)	480 (80.7)	560 (80.3)	579 (81.2)	
Other^b^	144 (7.2)	41 (6.9)	52 (7.5)	51 (7.2)	
Hispanic ethnicity	592 (29.5)	177 (29.7)	209 (30.0)	206 (28.9)	.89
Employed full or part time	639 (31.9)	154 (25.9)	234 (33.6)	251 (35.2)	<.001
Cause of ESKD					
Diabetes	964 (48.1)	269 (45.2)	332 (47.6)	363 (50.9)	<.001
Hypertension	332 (16.6)	123 (20.7)	106 (15.2)	103 (14.4)	
Glomerulonephritis	340 (17.0)	97 (16.3)	<130^c^	<120^c^	
Other	325 (16.2)	91 (15.3)	105 (15.1)	129 (18.1)	
Unknown	44 (2.2)	15 (2.5)	<30^c^	NA^c^	
Comorbid conditions^d^					
Hypertension	1751 (87.4)	523 (88.0)	613 (87.9)	615 (86.3)	.53
Diabetes	1060 (52.9)	322 (54.1)	365 (52.4)	373 (52.3)	.77
Congestive heart failure	273 (13.6)	99 (16.7)	79 (11.3)	95 (13.3)	.02
Atherosclerotic heart disease	139 (6.9)	57 (9.6)	43 (6.2)	39 (5.5)	.009
Peripheral vascular disease	109 (5.4)	40 (6.7)	39 (5.6)	30 (4.2)	.13
Chronic obstructive pulmonary disease	61 (3.0)	18 (3.0)	22 (3.2)	21 (2.9)	.97
Cerebrovascular disease/TIA	88 (4.4)	36 (6.1)	35 (5.0)	17 (2.4)	.003
Cancer	71 (3.5)	11 (1.9)	33 (4.7)	27 (3.8)	.02
Pre-ESKD nephrological care					
Yes	1306 (65.1)	360 (60.5)	457 (65.6)	489 (68.6)	<.001
No	500 (24.9)	183 (30.8)	171 (24.5)	146 (20.5)	
Unknown	199 (9.9)	52 (8.7)	69 (9.9)	78 (10.9)	
BMI, mean (SD)	29.0 (7.8)	28.8 (7.4)	29.0 (7.9)	29.2 (8.1)	.86
eGFR, mL/min/1.73m^2^, mean (SD)	9.4 (7.4)	9.3 (5.0)	9.2 (6.6)	9.6 (9.5)	.56
Market-level characteristics, median (IQR)					
Per-capita income, $	51 978 (43 348-58 064)	47 796 (37 128-55 342)	51 978 (43 348-58 064)	52 700 (46 145-62 492)	<.001
Dialysis market competition^e^	0.2 (0.0-0.4)	0.2 (0.0-0.6)	0.2 (0.0-0.4)	0.2 (0.0-0.4)	<.001

Abbreviations: BMI, body mass index (calculated as weight in kilograms divided by height in meters squared); eGFR, estimated glomerular filtration rate; ESKD, end-stage kidney disease; NA, not available; TIA, transient ischemic attack.

^a^ Other/no documented insurance is a combination of the 2 individual categories, where no documented insurance describes those with no observed record of medical insurer/payer in Colorado All Payer Claims Database (ie, all-time, specific year, and/or only with dental coverage) and other describes individuals who had a dialysis claim or an enrollment record (but no dialysis claim) in the relevant time period.

^b^ Other race includes American Indian or Alaska Native, Asian, Hawaiian or Pacific Islander, and other race. These categories were grouped together due to small sample sizes.

^c^ Some cells are masked due to small sample sizes.

^d^ Comorbid conditions of drug dependence, tobacco use, inability to ambulate, and inability to transfer were included as covariates but suppressed in this table. These conditions did not differ meaningfully by time period.

^e^ Dialysis market competition was operationalized by the Herfindahl-Hirschman Index of market competition, based on the number of patients undergoing dialysis unique to each facility in a hospital service area. Herfindahl-Hirschman Index values range from 0, reflecting a market with perfect competition, to 1, which indicates a monopolistic market.

Among individuals who were not enrolled in Medicare at ESKD onset, 883 (44.0%) were enrolled in Medicaid, 369 (18.4%) were enrolled in private insurance, and 753 (37.6%) had other or no documented insurance (Table 1). The proportion of those enrolled in private insurance at the time of dialysis initiation increased over time (91 of 595 patients [15.3%] in the pre-ACA period vs 144 of 713 patients [20.2%] in the later post-ACA period). Similarly, a greater proportion of patients new to dialysis had Medicaid coverage over time (209 of 595 patients [35.1%] in the pre-ACA period vs 355 of 716 patients [49.8%] in the later post-ACA period).

### Medicare Enrollment and Other Insurance in the Year After Dialysis Initiation

Over the entire observation period, of the sample’s 2005 patients, 1172 (58.5%) enrolled in Medicare during the first year after dialysis initiation, with most enrolling on day 1 of dialysis (Table 1, Table 2, and eFigure 2 in Supplement 1). Medicare enrollment on day 1 declined over the 3 time periods, from 392 of 595 patients (65.9%) in 2012 to 2013 to 312 of 713 patients (43.8%) in 2016 to 2017 (Table 1). Overall, non-Medicare coverage increased from 154 of 595 patients (25.8%) in the pre-ACA period to 279 of 697 patients (40.0%) and 284 of 713 patients (39.8%) in the 2014 to 2015 and 2016 to 2017 post-ACA years, respectively (eFigure 2 and eTable 2 in Supplement 1).

**Table 2.** New Medicare Enrollment 1 Year After Dialysis Initiation by Baseline Insurance Status and Dialysis Initiation Year (N = 2005)^a^

Insurance status	Pre-ACA, 2012-2013		Post-ACA period 1, 2014-2015		Post-ACA period 2, 2016-2017	
	Dialysis initiation, No. (column %)	Medicare by day 365, No. (row %)	Dialysis initiation, No. (column %)	Medicare by day 365, No. (row %)	Dialysis initiation, No. (column %)	Medicare by day 365, No. (row %)
Total patients	595 (100)	420 (70.6)	697 (100)	391 (56.1)	713 (100)	361 (50.6)
Medicaid	209 (35.1)	144 (68.9)	319 (45.8)	185 (58.0)	355 (49.8)	194 (54.6)
Private	91 (15.3)	62 (68.1)	134 (19.2)	70 (52.2)	144 (20.2)	66 (45.8)
Other/no documented insurance^b^	295 (49.6)	214 (72.5)	244 (35.0)	136 (55.7)	214 (30.0)	101 (47.2)

Abbreviation: ACA, Affordable Care Act.

^a^ The table displays the proportion of individuals who newly enrolled in Medicare among those with specific baseline insurance identified in the Colorado All Payer Claims Database.

^b^ Other/no documented insurance is a combination of the 2 individual categories, where no documented insurance describes those with no observed record of medical insurer/payer in the Colorado All Payer Claims Database (ie, all-time, specific year, and/or only with dental coverage) and other describes individuals who had a dialysis claim or an enrollment record (but no dialysis claim) in the relevant time period.

Unadjusted rates of switching from Medicaid to Medicare 1 year after dialysis initiation decreased 14.3 percentage points over time (68.9% pre-ACA vs 58.3% and 54.6% in the post-ACA periods, respectively; Table 2). Unadjusted rates of switching from private insurance to Medicare 1 year after dialysis initiation decreased by 22.3 percentage points (68.1% pre-ACA vs 52.2% and 45.8% in the post-ACA periods, respectively).

In adjusted analysis (eTable 3 in Supplement 1), individuals initiating dialysis in the initial post-ACA period (2014-2015) and the late post-ACA period (2016-2017) were less likely to enroll in Medicare (post-ACA period: risk ratio, 0.83; 95% CI, 0.76-0.91; late post-ACA period: risk ratio, 0.77; 95% CI, 0.70-0.84) than individuals initiating dialysis in the pre-ACA period (2012-2013). Medicare enrollment was similar between patients who had Medicaid or private insurance at dialysis initiation.

### Dialysis Spending in the First Year of Dialysis

Over the entire 2012 to 2017 period, quarterly dialysis spending in the first year of dialysis among patients with private insurance was higher than among those with Medicare coverage ($26 351-$29 781 vs $10 039-$12 741) and higher than those with Medicaid (Table 3). Quarterly dialysis spending fluctuated throughout the year for individuals with Medicaid, private, and unknown or no documented coverage and was stable for those with Medicare (Figure).

**Table 3.** Total Dialysis Spending by Selected Patient Insurance Status in the Year After Dialysis Initiation

Insurance status^a^	No. of patients	Total dialysis spending, median (IQR), $^b^
Medicare enrollment by end of year^c^		
Other/no documented insurance to Medicare^d^	238	27 349 (18 556-33 537)
Medicaid to Medicare	445	43 340 (34 184-53 686)
Private to Medicare	164	85 041 (59 172-161 703)
No Medicare enrollment by end of year		
Medicaid to other/no documented insurance^d^	75	14 493 (4310-27 859)
Medicaid remains Medicaid	182	38 710 (22 862-58 315)
Private remains private	68	111 374 (89 282-151 622)
Other/no documented insurance remains other/no documented insurance^d^	151	147 141 (99 253-258 821)

^a^ Reflects insurance status at the start of the quarter.

^b^ Dialysis spending includes patient and insurance costs.

^c^ Medicare costs from the Colorado All Payer Claims Database include patient and payer (including Medicare supplemental coverage) documented spending.

^d^ Other/no documented insurance is a combination of the 2 individual categories, where no documented insurance describes those with no observed record of medical insurer/payer in the Colorado All Payer Claims Database (ie, all-time, specific year, and/or only with dental coverage) and other describes individuals who had a dialysis claim or an enrollment record (but no dialysis claim) in the relevant time period.

**Figure.**
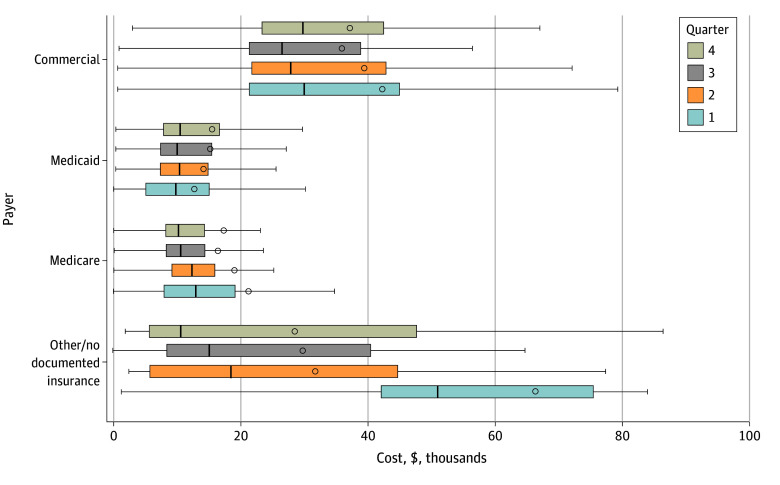
Median Quarterly Dialysis Spending After Dialysis Initiation by Insurance Type at Start of Quarter Cost data were generated from a subsample of 1095 patients who had a documented dialysis claim for the initiation of dialysis either in Medicare fee-for-service claims or in the Colorado All Payer Claims Database (CO APCD). Considering the sampling criteria of no prior Medicare enrollment, quarter 1 spending for new Medicare enrollees would be for patients receiving home dialysis (training). This is the 1 condition where the 90-day waiting period for new Medicare enrollment was waived. Medicare costs from the CO APCD include patient and payer (including Medicare supplemental coverage) documented spending. Other/no documented insurance is a combination of the 2 individual categories, where no documented insurance describes those with no observed record of medical insurer/payer in the CO APCD (ie, all-time, specific year, and/or only with dental coverage) and other describes individuals who had a dialysis claim or an enrollment record (but no dialysis claim) in relevant time period. Expenditures in this insurance category had to have a documented first dialysis claim at the start of quarter 1 but not necessarily for quarters 2 through 4 (ie, some dropped coverage and then resumed at some point in the quarter either to other or 1 of the other categories or had a self-pay claim). In this graph of dialysis spending, the range of values is depicted by whiskers, the boxes reflect the IQRs, the lines within each box indicate the median value, and circles indicate the mean value in quarterly dialysis spending.

Patient-level spending for selected patterns of insurance coverage was also examined because most patients in the sample switched insurance during their first year receiving dialysis (Table 3). Individuals who switched from private insurance to Medicare by the end of their first year incurred twice as much spending than those starting dialysis with Medicaid coverage (median [IQR], $85 041 [$59 172-$161 703] vs $43 340 [$34 184-$53 686]). For those who kept their private insurance coverage for 1 year after dialysis initiation, median (IQR) expenditures were higher than for those who remained enrolled in Medicaid 1 year after dialysis initiation ($111 374 [$89 282-$151 662] vs $38 710 [$22 862-$58 315]) and for those who transitioned from Medicaid to other or no documented coverage ($147 131 [$99 253-$258 821] vs $38 710 [$22 862-$58 315]).

## Discussion

ACA legislation has improved insurance access for many individuals in the US since its implementation in 2014, which has been particularly valuable for previously uninsured patients.^13,14^ For patients with kidney failure who are generally eligible for coverage via Medicare, the ACA expanded access to private insurance by barring plans from coverage denials due to preexisting conditions, promoting state insurance exchanges, and relaxing special enrollment periods.^15^ For organizations providing dialysis, patients’ expanded access to private insurance afforded opportunities to receive favorable dialysis payments than would be paid by Medicare. Improved access to insurance, combined with higher payments from private insurers, resulted in more patients remaining enrolled in private insurance, even if they were eligible to switch to Medicare.

We found the same to be true for individuals initiating dialysis who were enrolled in Medicaid at dialysis initiation. Colorado expanded its Medicaid program in 2014. Patients enrolled in Medicaid pay no premiums and have minimal cost-sharing requirements, so they have little incentive to voluntarily seek additional coverage. This may explain the low Medicare enrollment trends we observed in Colorado Medicaid beneficiaries.

We were able to examine insurance transitions after dialysis initiation because the unique linked data in this study provided granular details about insurance coverage to evaluate the hypothesis that reductions in Medicare enrollment coincided with greater enrollment in private or other insurance. The evidence from Colorado is consistent with previously reported national trends^5^ that relied on less granular data and align with policymaker concerns that patients are being diverted from Medicare to non-Medicare insurance.^15^ There is no national data source that allows the kind of insurance explorations that we evaluated in Colorado. Such linkage is needed to examine whether coverage trends of patients initiating dialysis observed herein are found in the other 49 states.

These findings are also consistent with prior reports that annual dialysis spending is higher among individuals with private coverage than Medicare. Previous analyses were based on estimated payments from corporate annual reports^16^ or data from private insurance claims^17,18^ and did not account for changes in coverage among patients initiating dialysis. As such, we present, to our knowledge, the first evidence to date that quantifies the extent of dialysis spending across patterns of insurance for patients in this critical first year after dialysis initiation, when decisions about treatment, coverage, and spending often extend into the long term.

Dialysis is an estimated $24.7 billion industry in the US^19^ and is the dominant treatment modality for 71% of the approximately 600 000 patients with ESKD. Patients with kidney failure are a clinically and demographically complex population, and their dependence on organizations providing dialysis for life sustaining care—whether dialysis or pursuing transplantation—cannot be overstated. As advances in treatment for kidney failure have stalled, strategies for organizational survival revolve around operational and clinical efficiencies, competitive advantage, and maximizing profits and revenues. While some of these strategies have been found to directly benefit patients, it has been unclear whether declining rates of Medicare enrollment affect patient care and national spending (private and public) on ESKD.

The dialysis industry is highly consolidated,^20^ so organizations providing dialysis are able to charge private insurers much higher rates than the typical Medicare reimbursement rate (annually $238 000 vs $80 000, respectively).^3^ This may partially explain why the share of privately insured patients increased modestly over the study period. Another potential and related factor is the advent of charitable premium assistance programs funded by organizations that provide dialysis that help patients acquire private insurance coverage while also generating considerably higher payments (and additional profit) for dialysis treatments.^15^ It is unclear how patients undergoing dialysis fare under charitable premium assistance and what happens to them when such assistance comes to an end. The impact of these charitable assistance programs is unclear because dialysis facilities are not required to report their patients’ sources of financing,^21^ but it has clear implications for Medicare and private insurance payment arrangements for dialysis and the cost burden to the health system.

As the US health care system transitions to value-based payment and care delivery, these findings also raise concerns about the implications of differential spending on the value of dialysis care. Dialysis expenditures for privately insured patients were at least twice as high as patients with Medicaid who enrolled in Medicare after dialysis initiation and several times higher for patients with Medicaid who did not enroll in Medicare. This may lead to unnecessarily high overall societal costs and inequitable access to care for some patients, which ultimately affects payers, patients, and overall consumers (ie, overall consumer health care insurance premiums are not limited to patients with ESKD).

Finally, these findings raise concerns about the effectiveness of federal policy to improve dialysis access, quality, and outcomes in the long term. As the historically dominant payer for individuals with kidney failure, Medicare has been a powerful lever for encouraging improvements in kidney care that benefit both Medicare and non-Medicare enrollees (eg, spillover effects of care in dialysis facilities or private payer incentive programs that often follow Medicare’s lead). Although many patients in 2012 to 2017 enrolled in Medicare within 1 year of dialysis initiation, the observed decline in Medicare enrollment signals fragmentation in payers for dialysis that may undermine Medicare’s dominant position as the primary payer for dialysis and its influence to nudge improvements in access, cost, and quality via policy changes. In this way, Medicare’s universal insurance coverage for individuals in the US with ESKD comes at substantial social cost but is also necessary for ensuring access and quality of life–sustaining treatment for all patients in the US with kidney failure.

### Limitations

These findings should be taken in the context of study limitations. First, these 2012 to 2017 data from the initial years of the ACA Marketplace rollout in Colorado may not reflect dialysis practice, spending, and insurance coverage in more recent years or in other states. For example, Medicare promoted practice improvements in less costly home dialysis and transplant after this study period that this analysis cannot address.^22^ Second, these results are based on traditional Medicare data, so we cannot speak to the experience of Medicare Advantage enrollees who are a growing number of all patients with ESKD.^23^ Our assessment of Medicare dialysis spending was agnostic to its role as primary or secondary payer and does not speak to patients’ transitions from private to Medicare coverage. We also lacked more granular data about insurance coverage among the 37% of Colorado patients with other or no documented insurance at dialysis initiation. As more payers join the CO APCD, this fraction of patients may decline over time. Last, despite attempts to adjust for factors to minimize bias, unmeasured confounding may exist and limits our ability to make causal claims.

## Conclusions

This cross-sectional study shows that as dialysis payer mix moves toward higher proportions of patients covered by non-Medicare sources, it will be important to understand the implications on health systems and patient outcomes. In this way, the present findings underscore a need for more comprehensive data to examine Medicare, Medicaid, and private insurance enrollment trends over time. These linkages are needed to comprehensively assess the consequences of federal policies on payers, organizations providing dialysis, physicians, and patients, and inform oversight of health insurance coverage and clinical practice.

## Notes

Supplement 1. **eFigure 1.** Cohort Sampling Scheme **eTable 1.** Baseline characteristics of CO patients initiating dialysis by whether they were already age-eligible or enrolled in Medicare at dialysis (excluded from cohort) or unenrolled (included in cohort) **eFigure 2.** Sankey diagram: New Medicare enrollment during first year of dialysis, by baseline insurance status and year of dialysis initiation **eTable 2.** Tabled data from eFigure 2 Sankey diagram **eTable 3.** Adjusted Results: Enrollment in Medicare by 365 days after dialysis initiation, end of follow-up, or death (n=2,005)

Supplement 2. Data Sharing Statement
