# Supplementary material for: Medicare Enrollment and Spending Among Patients Initiating Dialysis After the Affordable Care Act
Source: JAMA Health Forum. 2024 Dec 6;5(12):e244304. doi: 10.1001/jamahealthforum.2024.4304 (PMC11624581; doi:10.1001/jamahealthforum.2024.4304)
Supplement: Supplement 1. — eFigure 1. Cohort Sampling Scheme eTable 1. Baseline characteristics of CO patients initiating dialysis by whether they were already age-eligible or enrolled in Medicare at dialysis (excluded from cohort) or unenrolled (included in cohort) eFigure 2. Sankey diagram: New Medicare enrollment during first year of dialysis, by baseline insurance status and year of dialysis initiation eTable 2. Tabled data from eFigure 2 Sankey diagram eTable 3. Adjusted Results: Enrollment in Medicare by 365 days after dialysis initiation, end of follow-up, or death (n=2,005) [file jamahealthforum-e244304-s001.pdf]

Supplemental Online Content

Wang V, Wilson LE, Rowen NP, Sloan CE, Maciejewski ML, Hammill BG. Medicare enrollment and spending among patients initiating dialysis after the Affordable Care Act. *JAMA Health Forum*. 2024;5(12):e244304. doi:10.1001/jamahealthforum.2024.4304

eFigure 1. Cohort Sampling Scheme
eTable 1. Baseline characteristics of CO patients initiating dialysis by whether they were already age-eligible or enrolled in Medicare at dialysis (excluded from cohort) or unenrolled (included in cohort)
eFigure 2. Sankey diagram: New Medicare enrollment during first year of dialysis, by baseline insurance status and year of dialysis initiation
eTable 2. Tabled data from eFigure 2 Sankey diagram
eTable 3. Adjusted Results: Enrollment in Medicare by 365 days after dialysis initiation, end of follow-up, or death (n=2,005)

This supplemental material has been provided by the authors to give readers additional information about their work.

© 2024 Wang V et al. *JAMA Health Forum.*

eFigure 1. Cohort Sampling Scheme

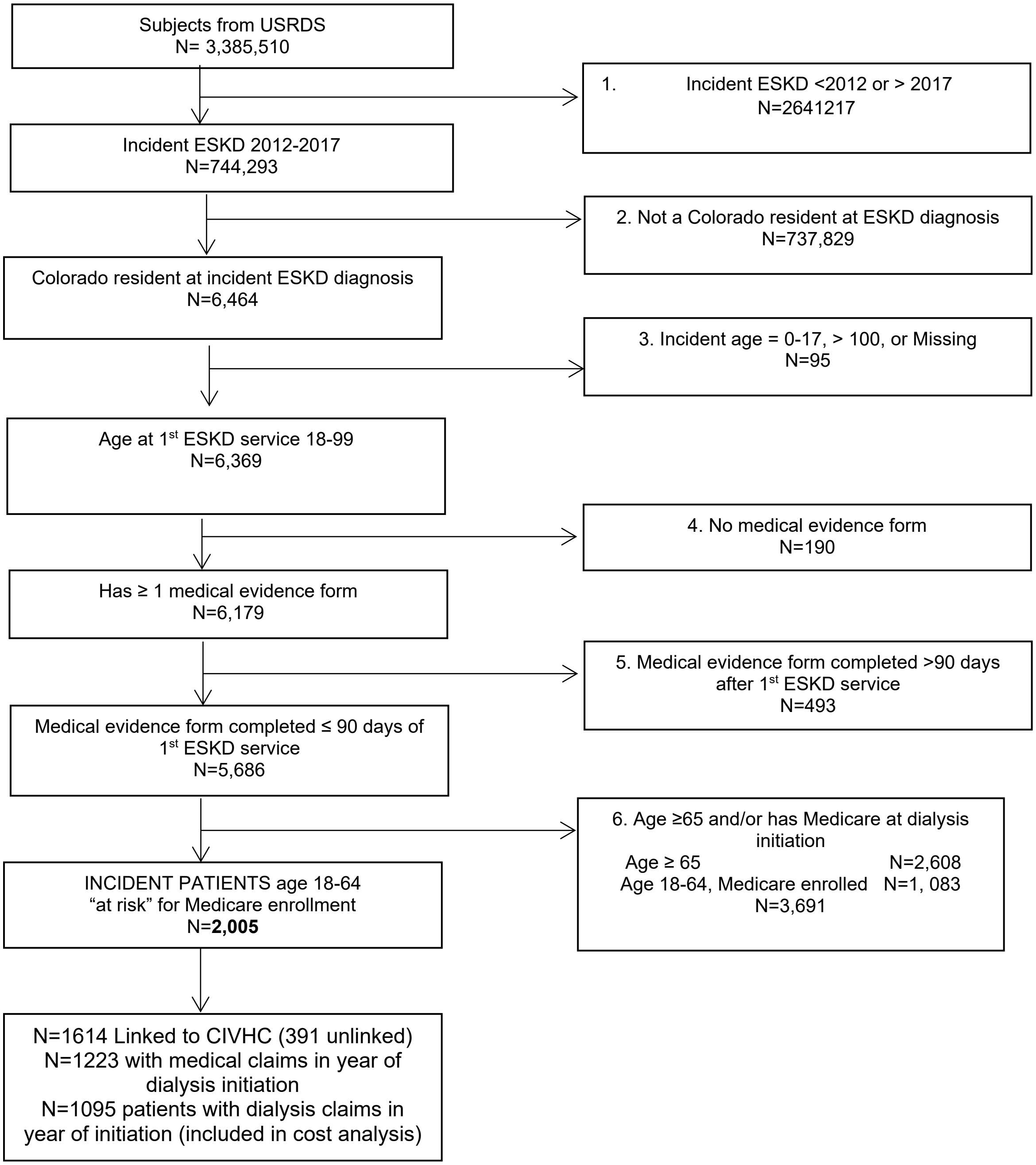

Notes:

1. A data linkage was conducted between the USRDS and the Colorado All-Payer Claims Data custodians (via data use agreement coordinated by – but not directly involving – this study team). This process involved identifying residents of Colorado who appeared in both the USRDS and Colorado APCD datasets, based on a minimum match of name and date of birth, and social security number, when available.
2. See Supplemental eTable 1 for descriptive statistics characterizing Colorado patients included for analysis (n=2005) and excluded from the sample due to age-eligibility and active enrollment in Medicare (n=3691).

© 2024 Wang V et al. *JAMA Health Forum.*

eTable 1. Baseline characteristics of CO patients initiating dialysis by whether they were already age-eligible or enrolled in Medicare at dialysis (excluded from cohort) or unenrolled (included in cohort)

Variable	Unenrolled	Enrolled or age-eligible (excluded from cohort)	P-value
N	2,005	3,681	
Age			< .001
18-44	589 (29.4%)	166 (4.5%)	
45-54	597 (29.8%)	307 (8.3%)	
55-64	819 (40.8%)	600 (16.3%)	
65+	0 (0.0%)	2,608 (70.9%)	
Male	1,259 (62.8%)	2,234 (60.7%)	0.12
Race and Ethnicity			< .001
White	1,619 (80.7%)	3,218 (87.4%)	
Black	242 (12.1%)	305 (8.3%)	
Other	144 (7.2%)	158 (4.3%)	
Hispanic Ethnicity	592 (29.5%)	711 (19.3%)	< .001
Employed full or part-time	639 (31.9%)	375 (10.2%)	< .001
Cause of ESKD			< .001
Diabetes	964 (48.1%)	1,857 (50.4%)	
Hypertension	332 (16.6%)	691 (18.8%)	
Glomerulonephritis	340 (17.0%)	373 (10.1%)	
Other	325 (16.2%)	681 (18.5%)	
Unknown	44 (2.2%)	79 (2.1%)	
Comorbid Conditions[1]			
Hypertension	1,751 (87.4%)	3,209 (87.3%)	.91
Diabetes	1,060 (52.9%)	2,122 (57.6%)	< .001
Congestive heart failure	273 (13.6%)	929 (25.3%)	< .001
Atherosclerotic heart disease	139 (6.9%)	547 (14.9%)	< .001
Peripheral vascular disease	109 (5.4%)	381 (10.4%)	< .001
Chronic obstructive pulmonary disease	61 (3.1%)	377 (10.4%)	< .001
Cerebrovascular disease/TIA	87 (4.4%)	317 (8.8%)	< .001
Cancer	70 (3.6%)	348 (9.6%)	< .001
Drug dependence	36 (1.8%)	25 (0.7%)	< .001
Tobacco use	160 (8.2%)	222 (6.1%)	.004
Inability to ambulate	40 (2.0%)	149 (4.1%)	< .001
Inability to transfer	16 (0.8%)	68 (1.9%)	.002
Pre-ESKD nephrology care			< .001
Yes	1,306 (65.1%)	2,902 (78.8%)	
No	500 (24.9%)	572 (15.5%)	
Unknown	199 (9.9%)	207 (5.6%)	
BMI, Mean (SD)	29.0 (7.8)	28.7 (7.1)	.61
eGFR b, mL/mn/1.73m2, Mean (SD)	9.4 (7.4)	10.2 (5.7)	< .001
**Market-level characteristics (Hospital Service Area)**			
Per capita income	51,978 (43,348- 58,064)	50,336 (43,304-58,064)	.02
Dialysis market competition[2]	0.2 (0.0, 0.4)	0.2 (0.0, 0.5)	.002

Notes:

1. Comorbid conditions ascertained from the patient-level CMS Medical Evidence Form, which is required of all patients initiating care for end-stage renal disease, regardless of source of insurance.
2. Dialysis market competition was operationalized by the Herfindahl-Hirschman Index (HHI) of market competition, based on the number of dialysis patients unique to each facility in a hospital service area (HSA, Dartmouth Atlas). HHI values range from 0 reflecting a market with perfect competition and 1 which indicates a monopolistic market.

© 2024 Wang V et al. *JAMA Health Forum.*

eFigure 2. Sankey diagram: New Medicare enrollment during first year of dialysis, by baseline insurance status and year of dialysis initiation

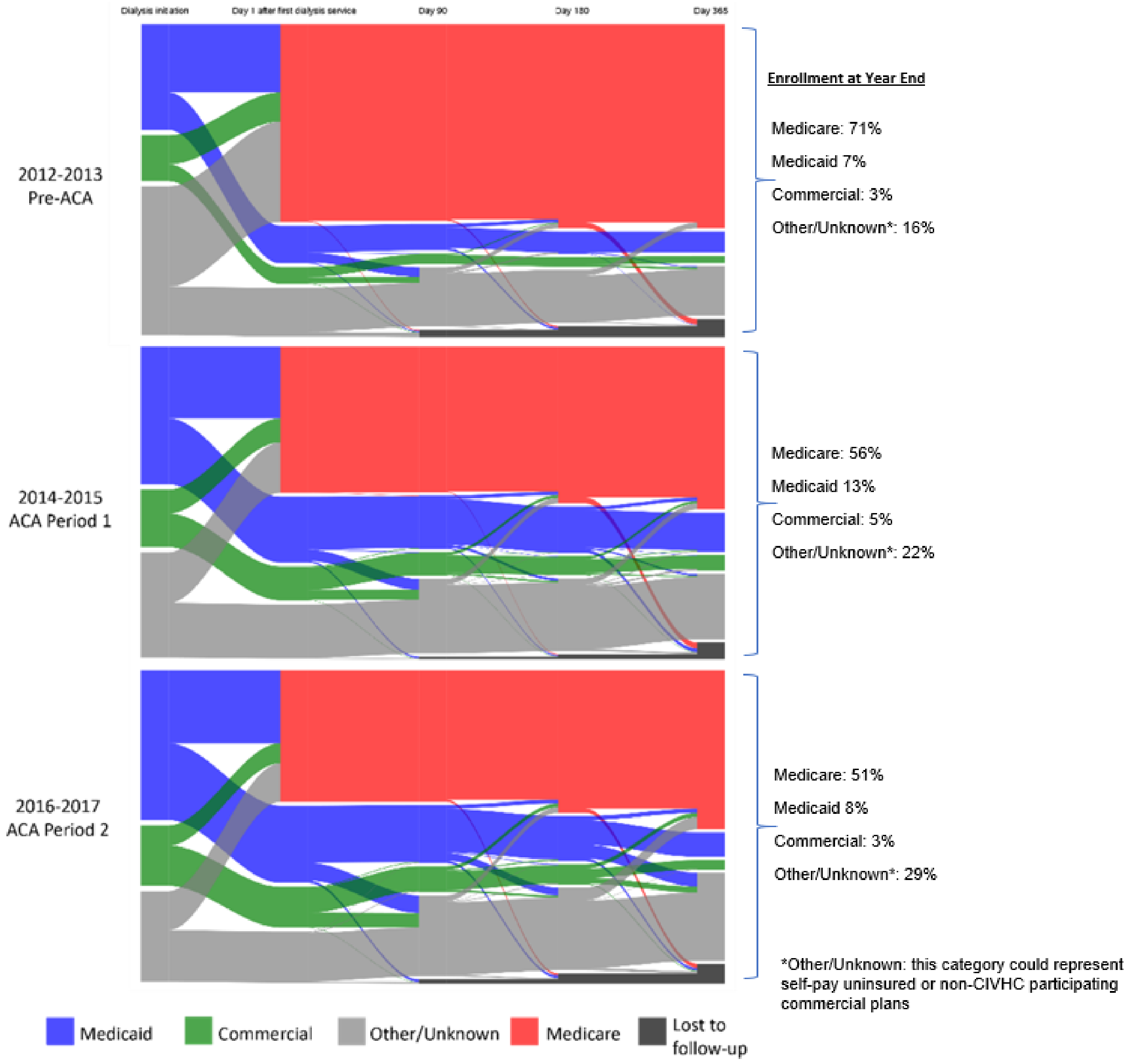

eTable 2. Tabled data from eFigure 2 Sankey diagram

	**Pre-ACA 2012-2013**		**Post ACA period 1 2014-2015**		**Post ACA period 2 2016-2017**		**Overall**	
**N**	595		697		713		2005	
**Time on Dialysis**	Initiation, N	Day 365, N (row %)	Initiation, N	Day 365, N (row %)	Initiation, N	Day 365, N (row %)	Initiation, N	Day 365, N (row %)
Medicare	--	422 (71)	--	390 (56)	--	365 (51)	--	1177 (59)
Non-Medicare								
Medicaid	209	42 (7)	319	91 (13)	355	57 (8)	883	190 (10)
Private	91	17 (3)	134	35 (5)	144	21 (3)	369	73 (4)
Other/Uknown insur	295	95 (16)	244	153 (22)	214	206 (29)	753	454 (23)
Lost to follow-up	--	19 (3)	--	28 (4)	--	64 (9)	--	111 (6)

Note: Figures reported in the text do not include individuals lost to follow-up.

© 2024 Wang V et al. *JAMA Health Forum.*

eTable 3. Adjusted Results: Enrollment in Medicare by 365 days after dialysis initiation, end of follow-up, or death (n=2,005)

Parameter	RR (95% CI)
Year of initiation (ref 2012-2013)	
2014-2016	0.83 (0.76-0.91)
2017-2018	0.77 (0.70-0.84)
Insurance at baseline (ref=Medicaid)	
Private	0.91 (0.81-1.01)
Other/No documented insurance	0.95 (0.87-1.03)
Age (ref 18-44)	
45-54	1.09 (0.99-1.21)
55-64	1.22 (1.11-1.35)
Male	1.08 (1.00-1.17)
Patient race (ref White)	
Black	1.06 (0.95-1.19)
Other	1.05 (0.91-1.21)
Hispanic ethnicity	0.94 (0.87-1.03)
Employed full or part-time	0.93 (0.85-1.01)
Cause of ESKD (ref Diabetes)	
Hypertension	1.03 (0.91-1.16)
Glomerulonephritis	0.96 (0.84-1.11)
Other	0.93 (0.81-1.07)
Unknown	0.69 (0.49-0.97)
Comorbid conditions	
Hypertension	1.10 (0.97-1.25)
Diabetes	1.02 (0.91-1.15)
Congestive heart failure	0.94 (0.85-1.05)
Atherosclerotic heart disease	1.03 (0.91-1.18)
Peripheral vascular disease	1.05 (0.91-1.21)
Chronic obstructive pulmonary disease	1.02 (0.84-1.23)
Cerebrovascular disease/TIA	0.97 (0.82-1.15)
Cancer	0.87 (0.69-1.10)
Drug dependence	0.42 (0.24-0.72)
Tobacco use	1.05 (0.92-1.20)
Inability to ambulate	0.95 (0.68-1.33)
Inability to transfer	0.83 (0.45-1.52)
Pre-ESKD nephrology care (ref = yes)	
No	1.11 (1.03-1.21)
Unknown	0.90 (0.78-1.05)
BMI	1.01 (1.00-1.01)
eGFR	1.00 (0.99-1.00)
HSA HHI	1.00 (0.87-1.15)
Per capita income	1.00 (1.00-1.00)

© 2024 Wang V et al. *JAMA Health Forum.*
