# Supplementary material for: Medicare Enrollment and Spending Among Patients Initiating Dialysis After the Affordable Care Act
Source: JAMA Health Forum. 2024 Dec 6;5(12):e244304. doi: 10.1001/jamahealthforum.2024.4304 (PMC11624581; doi:10.1001/jamahealthforum.2024.4304)
Supplement: Supplement 2. — Data Sharing Statement [file jamahealthforum-e244304-s002.pdf]

Data Sharing Statement

Wang. Medicare Enrollment and Spending Among Patients Initiating Dialysis After the Affordable Care Act. *JAMA Health Forum*. Published December 06, 2024. doi:10.1001/jamahealthforum.2024.4304

Data
Data available: No

Additional Information
Explanation for why data not available: The data used in this study are available from the US Renal Data System and the Center for Improving Value in Health Care with the execution of a data use agreement.
